# Enhancing task execution: a dual-layer approach with multi-queue adaptive priority scheduling

**DOI:** 10.7717/peerj-cs.2531

**Published:** 2024-12-03

**Authors:** Mansoor Iqbal, Muhammad Umar Shafiq, Shouzab Khan, Obaidullah, Saad Alahmari, Zahid Ullah

**Affiliations:** 1 Department of Electrical and Computer Engineering, University of Cyprus, Nicosia, Cyprus; 2 College of Arts and Sciences, University of Alabama - Birmingham, Birmingham, Alabama, United States of America; 3 Department of Computer Science, Northern Border University, Arar, Saudi Arabia; 4 Dipartimento di Elettronica, Informazione e Bioingeneria, Politecnico di Milano, Milan, Italy

**Keywords:** Job prioritization, Multi-queue threshold, Adjustable time quantum processing, Dynamic priority scheduling, Time-sensitive systems

## Abstract

Efficient task execution is critical to optimize the usage of computing resources in process scheduling. Various task scheduling algorithms ensure optimized and efficient use of computing resources. This article introduces an innovative dual-layer scheduling algorithm, Multi-Queue Adaptive Priority Scheduling (MQAPS), for task execution. MQAPS features a dual-layer hierarchy with a ready queue (RQ) and a secondary queue (SQ). New tasks enter the RQ, where they are prioritized, while the SQ contains tasks that have already used computing resources at least once, with priorities below a predefined threshold. The algorithm dynamically calculates the time slice based on process priorities to ensure efficient CPU utilization. In the RQ, the task’s priority level defines its prioritization, which ensures that important jobs are completed on time compared to other conventional methods where priority is fixed or no priority parameter is defined, resulting in starvation in low-priority jobs. The simulation results show that MQAPS better utilizes CPU resources and time than traditional round-robin (RR) and multi-level scheduling. The MQAPS showcases a promising scheduling technique ensuring a balanced framework for dynamic adjustment of time quantum and priority. The MQAPS algorithm demonstrated optimization, fairness, and efficiency in job scheduling.

## Introduction

Process scheduling is considered to be an integral part of operating systems and plays a pertinent role in computer systems to execute tasks and processes in an ordered fashion. The process is an instance of a program that requires system resources for execution. In the event of a new process creation, the operating system allocates system resources, and the scheduler adds them to a ready queue. Therefore, the scheduling algorithm defines the execution order of the process and executes the process. In this regard, various scheduling algorithms are discussed in the literature to ensure the timely execution of all assigned processes. Therefore, this article presents an innovative scheduling algorithm, Multi-Queue Adaptive Priority Scheduling (MQAPS), that utilizes the Round Robin (RR) with Adaptive Priority Scheduling (RRAPS) (Iqbal et al., 2023) scheduling method. The detailed working flow of the RRAPS algorithm is provided in Iqbal et al. (2023).

MQAPS incorporates a multiple-tiered structure, such as a ready queue (RQ) where tasks arrive initially and there is a further secondary queue (SQ) excluded or removed to supplementary SQ where the tasks experienced execution cycles and their priority has dropped below a given threshold, implemented so that algorithms may increase adaptation by dynamically altering time slices according to task execution and priorities thus avoiding starvation of resources. MQAPS speeds up the completion of high-priority jobs by biasing them, such that they are given priority for execution. Per task execution timings and priorities, the MQAPS changes dynamically per time quantum (TQ) + Priority. Dynamic TQ Scheduling is a twist of the classic RR approach, where it gives to each activity a quantum regarding its behavior.

The multi-queue (Shafi et al., 2020; Zhang et al., 2023; Chen & Jia, 2022) applied several traditional scheduling methods in separate queues. Algorithms such as first-come, first-served, shortest job first, RR, and priority scheduling (Bibu & Nwankwo, 2019). The MQAPS provides a two-level algorithm with great precision for objectives such as efficiency, fairness, and high priority in the RR scheduling. To implement an MQAPS, the target is to reduce system overhead and execution time of tasks as well. You do this by carefully planning your task scheduling and resource allocations without adding any additional complexity. We further strengthen the argument for MQAPS by showing through extensive testing and analysis that it can maintain minimal system overhead under varying workloads and conditions. Based on selected tasks, the performance of MQAPS and the results are compared with RR scheduling. Since it demonstrates substantial improvements in both execution and resource consumption, a valid way to execute tasks could be MQAPS. MQAPS’s unique way of modifying priorities and time quantum plays a substantial role in efficient recourse utilization and achieves fairness (fair allocation of CPU time among tasks) as compared to RR conventional methods and contributes significantly to the existing literature on scheduling methods without explicitly referencing the RRAPS algorithm (Iqbal et al., 2023).

To address the issue of starvation and ensure fairness, both the methods *i.e.,* RRAPS and MQAPS share a fundamental methodology for process scheduling by adjusting time quantum (TQ) and process priorities dynamically. In both methods, resource allocation is optimized by adjusting TQ, which is calculated based on the mean burst time of all the processes in the queue. Additionally, they both reinsert processes that do not complete within their assigned TQ back into a queue with adjusted priorities, ensuring that processes continue to progress toward completion. This adaptive priority management is central to both RRAPS and MQAPS, as it balances the need for efficiency with the necessity of preventing any single process from monopolizing CPU time. Despite these shared principles, MQAPS extends the concept by introducing a dual-queue structure, which further refines the scheduling process and resource management.

The key differences between RRAPS and MQAPS lie in their queue structures and process handling. RRAPS uses a single ready queue (RQ) for all processes, while MQAPS employs a dual-queue system with a ready queue (RQ) and a secondary queue (SQ). This allows MQAPS to better manage processes by moving lower-priority tasks to the SQ, thus ensuring higher-priority tasks in the RQ are executed more efficiently. Additionally, when the RQ is empty, MQAPS shifts focus to the SQ, unlike RRAPS, which only operates within the RQ. MQAPS terminates only when both queues are empty, whereas RRAPS concludes when the RQ is completed. This hierarchical and more granular approach in MQAPS results in more refined and efficient scheduling compared to RRAPS’s simpler structure.

The rest of the article is presented in different sections: the literature review section reviews the current literature on the MQAPS scheduling algorithm. The methodology section discusses the proposed solution and its effectiveness. The performance validation section discusses the MQAPS algorithm’s outcomes. The conclusion and future directions provide concluding remarks.

## literature Review

The literature showcases the different scheduling methods and systems, focusing on multi-level, period, and prioritization techniques. For example, in Sharma et al. (2022), the authors presented a method to compute the time quantum, *i.e.,* Time quantum = (ProcessesAverage + BurstTimeMedian)/2. This method assigns the time quantum to the process at the front of the ready queue and is repeated with the entry or exit of a new process from RQ, providing a new TQ for the execution of processes. The authors in Mostafa & Amano (2020) described a revolutionary RR technique that capitalized on the benefits of prioritizing processes with short burst time (BT) while minimizing system performance and overhead. CPU-based processes are organized in the same group and share the same TQ. This novel method reduced average waiting and turnaround time and non-context switch overhead (NCS). Moreover, a CPU-based algorithm is introduced in Chandiramani, Verma & Sivagami (2019) with the main feature of preemption based on priority as well as the aging property to prevent starvation. In Ali, Marikal & Anil Kumar (2020), the author presented a hybrid round-robin scheduling mechanism by using dynamic time quantum, average. waiting and turnaround, and response time, as well as system overhead, is reduced. The round robin, shortest job first (SJF), and first come, first serve (FCFS) algorithms’ characteristics are all inherited by this strategy. One of the upgrades and modifications to the round-robin scheduling algorithm is the novel strategy known as VORR (variant on round-robin) discussed in Abdelhafiz (2021), which is covered in this work. Establishing an effective time quantum based on the median of burst times efficiently utilizes the CPU. The testing results have shown the value of the suggested technique in terms of average waiting time, average turnaround time, and context switches. Additionally, it improves various RR algorithms’ response times.

In the round-robin with adaptive priority scheduling (RRAPS) algorithm (Iqbal et al., 2023), the CPU runs the highest-priority process from the RQ. If the RQ is empty, the TQ corresponds to the process’s BT; otherwise, it represents the average BT of all RQ processes. If a process does not complete inside its TQ, its priority is reduced by one (if greater than 1), and it returns to the RQ. This cycle will continue until all processes are completed. The authors in Ghazy et al. (2022) devised a method that combined the RR, mean, and median. To determine a suitable dynamic TQ, the BT is calculated using the median and mean of the program instances in each group. The proposed method led to significant reductions in CPU overhead and utilization. In Alhaidari & Balharith (2021), the authors automated updating the TQ based on the task’s average TQ and remaining BT. Experiment results indicate that competing algorithms are outperformed in terms of latency turnaround time and response time. Moreover, Gupta et al. (2021) proposed a method called Optimized Round Robin (ORR) that minimized average waiting time, turnaround time, and the number of context switches while maximizing system throughput. This approach was created using machine learning and a trained model to estimate the best TQ value. ORR regularly outperforms RRSA and five upgraded versions of RRSA in maximizing performance within time-sharing operating systems in experimental comparisons. The suggested ORR algorithm produces superior results and opens up an avenue for equitable work scheduling on computers and real systems. Noon, Kalakech & Kadry (2011) described AN Algorithm, which uses a dynamic-time-quantum technique to update the time quantum based on the burst times of processes in the ready queue. Our simulations demonstrate that this strategy overcomes the restrictions of fixed-time quantum and increases RR performance. A variety of RR scheduling strategies have been explored, including smallest job first, preemption or non-preemption, and come first served first algorithms have been investigated in Omar, Jihad & Hussein (2021), Ali et al. (2021), Omotehinwa (2022) and Vecliuc, Leon & Logofătu (2022) to boost performance, usage, and throughput of CPU while minimizing system overhead.

The authors in Kim, Kim & Luh (2019) proposed a scheduling algorithm based on the round-robin heuristic *via* numerous work queues that outperformed the standard First-in-First-Out heuristic utilized by current platforms. The proposed algorithm was tested through an integration process, and statistical approaches confirmed the results. In Rafi et al. (2018), the proposed approach improved operating system functionality by establishing multiple queues for various process priorities and orchestrating RR queue scheduling with dynamic time slicing. Based on user-defined or system-defined criteria, processes are assigned to specific queues. Several case studies demonstrate the system’s usefulness, and the results are compared to those achieved using a priority scheduling approach. Zhao et al. (2019) introduced a new approach called the preemptive multi-queue fair queuing technique (P-MQFQ). The P-MQFQ efficiently dispatched threads from various programs based on CPU bandwidth from many cores, improving utilization and performance for parallel tasks in Linux and Xen. In Manuel et al. (2019), the authors used strategies to improve the Fittest Job First Dynamic Round Robin (FJFDRR) by leveraging a dual queue and using the process arrival time as an algorithmic element. The performance of the proposed approach, dubbed enhanced Fittest Job First Dynamic Round Robin (eFJFDRR) and the FJFDRR algorithm, was then compared to that of the other CPU scheduling algorithms. The trial data revealed that the eFJFDRR scheduling algorithm performed better in terms of minimizing average waiting time, turnaround time, and reaction time in various situations. Further, the number of context switches made by the processor during execution might be balanced. Niu et al. (2023) discussed the Infinite Queue in One Queue (IQQ) structure with in-queue scheduling approaches and unique procedures to fulfill the demands of varied flows.

To address the challenges of optimizing system execution time in heterogeneous computing environments, recent research has focused on advanced co-scheduling strategies that integrate hardware–software resource allocation with real-time scheduling methods. One such approach proposes a novel strategy that balances the execution of parallel workloads across CPUs and FPGAs, utilizing a high-performance heuristic scheduling algorithm to minimize execution time (Xu, Shi & Chen, 2023). Complementing this, the development of AnTiQ, a hardware-accelerated priority queue, enables efficient timer queue management by performing key operations such as PUSH, POP, PEEK, and DROP in constant time, enhancing scheduling efficiency in embedded systems (Nurmi et al., 2023). Building on this, schedulers optimized for multi-core CPUs leverage the Earliest Deadline First (EDF) algorithm, enabling parallel execution of up to four threads while supporting task suspension, resumption, and inter-task synchronization. By employing hardware-accelerated priority queues, these schedulers can make scheduling decisions in just two clock cycles, independent of system load (Norollah et al., 2021). For real-time many-core systems, hardware schedulers further enhance performance by scheduling dependent tasks using EDF and grouping related tasks based on dependencies (Lukás˘ & Mach, 2023). Additionally, the HCoD (Hardware coexecution Dispatcher) facilitates transparent co-execution across heterogeneous SoCs, distributing workloads between CPU cores and GPU units while dynamically balancing the load to prevent performance bottlenecks (Perez & Bosque, 2024). Together, these developments underscore the critical role of hardware acceleration and sophisticated scheduling algorithms in optimizing system performance for both general-purpose and specialized computing tasks.

Compared to conventional techniques, the aforementioned studies investigated several round-robin scheduling algorithms to improve system performance. However, there is always a need for continuous efforts to provide new types of scheduling algorithms and enhance performance in certain circumstances. Within this framework, our work suggests a novel method to enhance process scheduling and execution. Our technique incorporates multi-queue, adjustable TQ, and priorities into the RR and introduces diminishing priorities. The detailed comparison is shown in Table 1.

**Table 1:** Comparison of various scheduling algorithms.

Methods	Key features	Improvements	Comparison to MQAPS
RRAPS (Iqbal et al., 2023)	Adaptive TQ and priority	Reduced system overhead	Adaptive TQ and priority, Multi-Queue
Sharma (Sharma et al., 2022)	Dynamic TQ Calculation	New TQ per process entry/exit	Dynamic TQ, dual queues, adaptive priorities
Mostafa (Mostafa & Amano, 2020)	Prioritizes short BT processes	Reduced avg. waiting/ turnaround	Optimizes with secondary queue
Chandiramani (Chandiramani, Verma & Sivagami, 2019)	Preemption, aging	Prevents starvation	Adds dynamic TQ, secondary queue
Khaji (Ali, Marikal & Anil Kumar, 2020)	Hybrid RR with dynamic TQ	Reduced avg. waiting/ turnaround	Integrates with multi-tiered structure
Abdelhafiz (Abdelhafiz, 2021)	VORR with effective TQ	Improved waiting, turnaround time	Dynamic TQ, dual queue, adaptive priorities
Ghazy (Ghazy et al., 2022)	Combined RR, mean, median TQ	Reduced CPU overhead	Dual queues, dynamic priorities
Alhaidari (Alhaidari & Balharith, 2021)	Automated TQ update	Better latency, turnaround	Dynamic TQ, priority-based queue
Gupta (Gupta et al., 2021)	Optimized RR with ML	Minimized waiting, turnaround	Dynamic priority adjustment, dual-queue
Noon (Noon, Kalakech & Kadry, 2011)	Dynamic TQ	Limitation of fixed TQ	Dual-queue, Adaptive priority and TQ
Kim (Kim, Kim & Luh, 2019)	RR with multiple queues	Outperformed FIFO	Multi-queue, dynamic priorities
Rafi (Rafi et al., 2018)	Multiple queues for priorities	Improved functionality	Refined dual-queue system, adaptive TQ
Zhao (Zhao et al., 2019)	P-MQFQ	Better utilization, performance	Execution efficiency, fairness
Manuel (Manuel et al., 2019)	eFJFDRR, dual queue	Better waiting, turnaround times	Dual-queue, dynamic priority adjustment
Niu (Niu et al., 2023)	IQQ structure	Met varied flow demands	Multi-tier queue for scheduling efficiency

This adaptive method improves resource utilization efficiency and guarantees key tasks are completed on time. Prioritization of higher-priority activities, which expedites their execution once they enter the RQ, is a distinguishing feature. Further, the proposed system incorporates a secondary queue for managing activities that passed through previous execution cycles and had their priority reduced below a predetermined threshold. The dynamic decrease of priority for unfinished high-priority processes is another innovation that keeps low-priority processes from starving. To the authors’ knowledge, the RR scheduling algorithm is the first to use a multi-tiered queue structure, priorities, diminishing priorities, and dynamic TQ. Our proposed methodology provides a fresh and thorough perspective to the current literature and promises to increase efficiency in process scheduling. Given the limitations of traditional multi-queue scheduling algorithms in balancing efficiency, fairness, and priority, the Multi-Queue Adaptive Priority Scheduling (MQAPS) algorithm offers an improved solution to address these challenges.

## Methodology

This article presents an MQAPS that dynamically adapts the periods based on the BT and priority of the processes in the RQ and SQ. The MQAPS tackles the TQ problem. The TQ is computed using a simple method that takes the average BT for operations in the queue. This TQ computation affects both the RQ and SQ. After selecting a process, the CPU runs it for the calculated time. If the procedure is not finished, its priority is reduced by one. Following that, the process is reviewed, and if its priority falls below or equals the predefined threshold, it is shifted to the secondary queue; otherwise, it returns to the ready queue. MQAPS distributes system resources equally. The following subsections define the proposed algorithm, pseudo code, and flowchart of the proposed algorithm.

### Proposed algorithm

The MQAPS (Multi-Queue Adaptive Priority Scheduling) algorithm is an advanced scheduling technique leveraging a multi-queue architecture. It operates with two distinct queues: a ready queue (RQ) and a secondary queue (SQ). Upon the arrival of a new process in the RQ, the scheduler selects the highest-priority process for execution from the RQ. If the RQ is non-empty, the time quantum (TQ) is computed as the average burst time (BT) of all processes within the RQ. Conversely, if the RQ is empty, the TQ is assigned as the BT of the selected process. Processes execute for the assigned TQ. Upon termination of the execution, the process is marked as completed. However, if a process is not completed and its priority exceeds 1, the scheduler decrements its priority by one. If the adjusted priority exceeds 2, the process remains in the RQ with an updated BT. Otherwise, the process is migrated to the SQ if the priority is reduced to 2 or below. When the RQ is exhausted, the scheduler switches to the SQ, selecting the highest-priority process for execution. In this case, the TQ is determined similarly: if there are processes in the SQ, it is set as the average BT of all SQ processes; otherwise, the TQ is set to the individual process’s BT. After execution in the SQ, if the process priority is 2, it is decreased to 1 and returned to the SQ; if it is already 1, it is returned to the SQ without further modification. Upon process completion, it is marked as finished. In summary, MQAPS dynamically adjusts process priority and Time Quantum based on queue conditions, ensuring efficient CPU utilization across varying workloads while balancing between high- and low-priority tasks. The algorithm terminates until both queues are empty. Some of the pertinent features of the MQAPS algorithm are as follows:

• Efficient Allocation for High-Priority Processes. The system will guarantee that critical processes execute as they arrive in RQ.
• Mitigation of Starvation through Adaptive Priority as low priority process will also have a fair share of CPU.
• Fair CPU Time Distribution Across Queues. Both queues have a decreased priority parameter will ensure the process execution.
• Compatibility and Seamless Integration into existing RR scheduling frameworks with minimal modifications.

**Figure 1:**
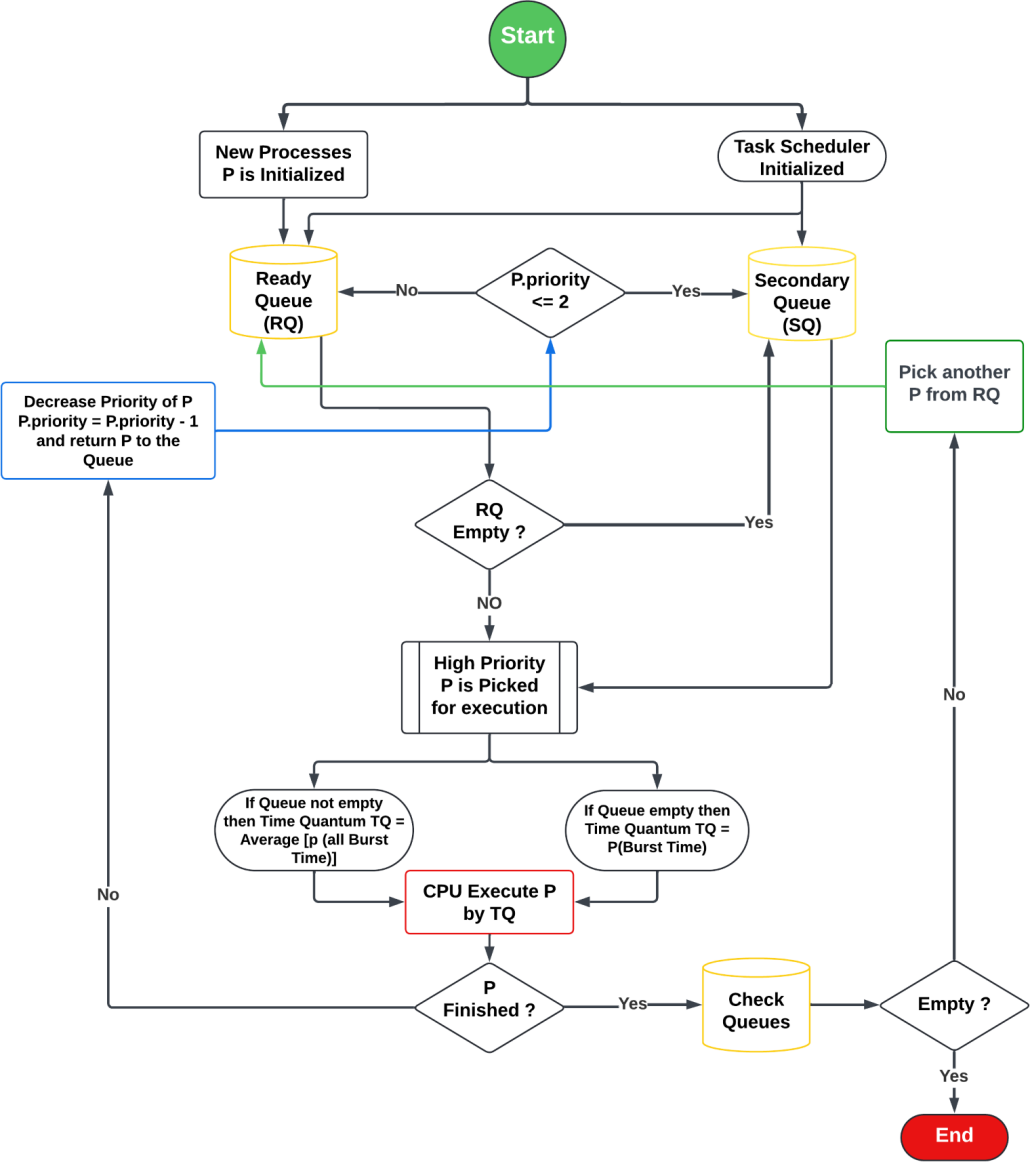
Flow chart of MQAPS algorithm.

The Multi-Queue Adaptive Priority Scheduling (MQAPS) algorithm operates by managing two critical queues: the RQ and the SQ, as illustrated in Fig. 1. The RQ holds newly arriving tasks, while the SQ contains previously executed tasks with priorities that have dropped below a specified threshold.

### Flow chart

Upon the arrival of a new process in the RQ, the CPU selects and executes the highest-priority task. If the RQ is initially empty, the TQ is set to the BT of the incoming process. If multiple processes are present in the RQ, the TQ is determined by averaging the burst times of all processes, ensuring a balanced CPU time distribution based on workload demands. The CPU executes the selected task for the assigned TQ. If the task is completed within this time frame, it is marked as finished. If not, the task’s priority is reduced by one. Should its priority drop to 2, the task is moved to the SQ. Otherwise, the task is returned to the RQ with an updated burst time.

When the RQ is empty, the scheduler switches to the SQ, executing the highest-priority task (as all processes in the SQ have priorities of 2 or lower). If the SQ is also empty, the TQ is set to the burst time of the selected process. If multiple tasks are present in the SQ, the TQ is calculated by averaging their burst times. The CPU then runs the selected task for the allocated TQ.

After execution, if the task’s priority is 2, it is reduced to 1 and re-queued in the SQ for further execution. If the task already has the lowest priority (1), it is re-added to the SQ without a priority change. This cycle continues until both the RQ and SQ are empty, signaling the end of the algorithm’s execution.

 
____________________________ 
Algorithm 1 Multi-Queue Adaptive Priority Scheduling (MQAPS)_______________________________________ 
  1:  Initialize ReadyQueue (RQ), SecondaryQueue (SQ) 
  2:  Initialization of new process P and enter it into RQ 
  3:  Scheduler picks high-priority P from RQ and loads it to CPU 
  4:  if RQ == ∅ then 
  5:       TQ = P _BT 
 6:  else 
  7:       TQ = Avg(BT _of_P′s_in_RQ) 
  8:  end if 
  9:  P executed by CPU for specified TQ 
10:  if P == completed then 
11:       P mark as job completed 
12:  end if 
13:  if P ⁄= completed then 
14:       if P.priority > 1 then // lowest priority is 1. 
15:            P.priority = P.priority − 1 
16:       end if 
17:       if P.priority <= 2 then 
18:            Shift to SQ with modified BT 
19:       else 
20:            Return to RQ with modified BT 
21:       end if 
22:       Repeat from Step 8 to 22 
23:  end if 
24:  if RQ == ∅ then 
25:       Load high-priority process from SQ to CPU for execution 
26:       if SQ == ∅ then 
27:            TQ = P _BT 
28:       else 
29:            TQ = Avg(BT _of_P′s_in_SQ) 
30:       end if 
31:       CPU start executing P by specified TQ 
32:       if P == completed then 
33:            P Mark as job completed 
34:       end if 
35:       if P ⁄= completed then 
36:            if P.priority > 1 then //1 is the lowest priority. 
37:                P.priority = P.priority − 1 
38:                Return to SQ with modified BT 
39:            else 
40:                Return to SQ with modified BT 
41:            end if 
42:       end if 
43:       Repeat Steps 24 to 43 
44:  end if  
45:  if RQ == ∅ && SQ == ∅ then 
46:       End Algorithm 
47:  else 
48:       Repeat Steps 3 to 48 
49:  end if_____________________________________________________________________________________________________________    

### Pseudo code

Algorithm 1 describes in full how the MQAPS algorithm operates. The process starts with the initialization of two important structures: the RQ and the SQ. When a new process enters the system, it is instantly added to the RQ, where the scheduler chooses the most important job for CPU execution. The TQ for this procedure is then calculated based on the RQ’s state. If the RQ is empty, the TQ is set to match the selected process’s BT, allowing it to operate continuously. However, if additional processes are waiting in the RQ, the TQ is dynamically adjusted to the average burst time of all queued processes, resulting in balanced and efficient CPU utilization. The CPU then executes the specified process for the given TQ, as detailed in steps 1–9.

When a process has finished its execution, it is marked as completed. If the process is not yet complete, its priority is reduced by one, indicating a lower urgency. If the priority is lower than 2, the process is routed to the SQ for further execution. This transfer to the SQ guarantees that lower-priority operations do not use all CPU resources, allowing higher-priority processes to be performed more efficiently. This procedure is controlled in a systematic manner, as stated in steps 10 through 22.

Steps 24 to 43 detail the procedure followed when the RQ is empty. In this scenario, the scheduler shifts focus to the SQ, selecting processes for execution. The TQ for these processes is dynamically calculated, similar to when processes are selected from the RQ. Once a process completes its execution, it is marked as completed. If a process has a priority level of 2, its priority is decreased by 1, and it is returned to the SQ for another execution cycle. If the process is already at the lowest priority level (priority 1), it is simply returned to the SQ without any change in priority. This iterative process of selecting, executing, and adjusting priorities continues seamlessly, ensuring that no process is left unattended until both the RQ and SQ are entirely emptied of processes. This mechanism ensures fairness and resource optimization, preventing low-priority processes from being indefinitely postponed.

Steps 45–49 deal with the finish of the scheduling process. If both the RQ and the SQ are empty, the algorithm finishes, indicating that all processes were completed. However, if each queue has any remaining processes, the algorithm will cycle through them, dynamically selecting and performing tasks. This guarantees that all operations are handled efficiently, with a focus on maintaining optimal resource utilization and good priority management.

## Performance validation

The MQAPS algorithm was tested on a 12th-generation Intel Core i7 CPU clocked at 2.40 GHz and equipped with 16 GB of RAM. To comprehensively compare MQAPS to the classic round-robin algorithm, a simulation environment was created using MATLAB 2022b. MATLAB was chosen because of its powerful programming tools and advanced graphical representation capabilities, which are required for effectively viewing and analyzing scheduling algorithms. Because MQAPS was tested *via* simulation, all required data—such as BT, priority levels, and process arrival times—were created programmatically within MATLAB, ensuring controlled and reproducible testing circumstances.

MQAPS, in contrast to RRAPS (Iqbal et al., 2023), presents an improved simulation model that takes a list as input containing the number of processes, time of arrival, burst times, and priority. The logic of our system uses the process’s execution history and priority to decide whether to add it to the ready or secondary queue. We define and set the priority reduction parameter to −1. When a process completes execution and its priority is greater than 1 (it cannot be less than 1), the process priority is dropped by one. This technique guarantees that non-critical processes get a fair share of the CPU.

The effectiveness and evaluation of the MQAPS algorithm employing four, five, and ten processes as a sample group with varying burst periods, arrival times, and priority are performed similarly to Iqbal et al. (2023). The suggested system’s robustness and efficacy are also tested using a broader sample of varied processes. The results are compared with Iqbal et al. (2023), Noon, Kalakech & Kadry (2011), and Rafi et al. (2018), as shown in the results and discussion section.

The simulation is used to test the MQAPS system. The simulation starts with the list_class by creating a process within the list_class, which includes adding and removing nodes from the list_class, along with computing turnaround and waiting times. In the list class, the Random() function is used to generate the burst time, priority, and arrival time using the InsertNodeMQAPS() method. In the second stage, a process is chosen by calling SelectProcessFromQueueMQAPS(), which chooses one of the two queues based on priority and execution history. When the process is chosen, the time quantum is computed using the TimeQuantumMQAPS() function. The ExecuteProcessMQAPS() method is used to execute the process for a specified time quantum. After completing a process, the ReturnTimeMQAPS() function calculates the waiting and turnaround time, and the processed process is removed using the DeleteNodeMQAPS() function. If the process is not terminated, the InsertNodeMQAPS() method is called once more. At the end of the simulation, the average turnaround time and average waiting time for all processes are calculated and published. This method ensures that MQAPS is extensively assessed in a simulated environment by providing important insights into performance measurements.

### Performance metrics

Certain metrics are crucial for evaluating and examining the performance of an algorithm. Some of these performance metrics that we used for the evaluation of MQAPS are as follows:

• Turnaround time: The time it takes for a process to complete its execution. The turnaround time can be calculated as Turnaround Time = Completion Time − Submission Time. The turnaround time is in milliseconds. The lesser the time, the better the turnaround time of the system
• Waiting time: Defines the duration and waiting time of a process in a queue before execution. Waiting time is calculated as Waiting Time = Execution Time − Submission Time. Where execution time is when the process enters the CPU. The waiting time is also measured in milliseconds. Less waiting represents the effectiveness and, again, property of the system.
• Context switching: This process can be characterized as the number of times a process switches from one to another and can also be termed system overhead. This process is time and resource-intensive, affecting system performance. The lesser the number of context switches, the lesser the system overhead.

A straightforward example is presented to illustrate the functionality of the MQAPS algorithm. This example highlights the advantages of MQAPS over the traditional RR algorithm, demonstrating how it more effectively handles task priorities and time quanta. Specifically, it showcases how MQAPS reduces both waiting and turnaround times compared to RR, emphasizing its efficiency in task management. Table 2 shows the process IDs, arrival and burst times, and priorities.

**Table 2:** Processes with arrival time, burst time and priority.

Process	Arrival time	Burst time	Priority (1: highest, 5: lowest)
P1	0	15	1
P2	1	10	3
P2	2	5	5

The working of the RR algorithm is as follows: Let us assume a time quantum (TQ) of 5 ms is used in RR scheduling.

• P1 arrives first and runs for 5 ms, leaving 10 ms of burst time.
• P2 arrives at *t* = 1 and runs for 5 ms, leaving 5 ms of burst time.
• P3 arrives at *t* = 2, runs for 5 ms, and completes its execution.
• At *t* = 4, P1 runs for another 5 ms and leaves 5 ms behind.
• At *t* = 5, P2 runs for another 5 ms and completes its execution.
• At *t* = 6, P1 runs for another round of 5 ms and completes its execution.

Table 3 illustrates the analysis of the RR algorithm.

**Table 3:** Comparative analysis of round robin having TQ = 5.

Process	Completion time (CT)	Turnaround time (TAT = CT-Arrival Time)	Waiting time (WT = TAT-BT)
P1	30	30 - 0 = 30	30 - 15 = 15
P2	25	25 - 1 = 24	24 - 10 = 14
P2	15	15 - 2 = 13	13 - 5 = 8

Now the working of MQAPS is as follows: The initial setup is given below.

• RQ: Holds newly arriving processes.
• SQ: Holds processes that have undergone execution and had their priority lowered to a threshold (≤2).

#### Step 1

P1 arrives at *t* = 0 and P1 enters the RQ at *t* = 0 with a burst time of 15 ms and the lowest priority (1). Since P1 is the only process in the queue, it is selected for execution. The time quantum (TQ) for P1 is set to its entire burst time (15 ms). Execution: P1 executes for its entire burst time of 15 ms, completing its execution at *t* = 15.

#### Step 2

P2 arrives at *t* = 1. While P1 runs, P2 arrives at *t* = 1 with a burst time of 10 ms and a priority of 3. P2 waits in the RQ.

#### Step 3

P3 arrives at *t* = 2, with a burst time of 5 ms and the highest priority (5). P3 is added to the RQ.

#### Step 4

P1 completes at *t* = 15, P1 completes its execution at *t* = 15, freeing up the CPU. The scheduler checks the RQ and finds P2 (priority 3) and P3 (priority 5) waiting. P3 has the highest priority (5), so it is selected for execution.

#### Step 5

P3 executes (*t* = 15 to *t* = 20). The scheduler sets the TQ for P3 to its entire burst time of 5 ms, as it is the highest priority process. P3 executes for 5 ms from *t* = 15 to *t* = 20, completing its execution.

#### Step 6

P2 executes (*t* = 20 to *t* = 30). After P3 finishes, P2 remains in the RQ. The scheduler sets the time quantum (TQ) for P2 to its entire burst time of 10 ms, as it is the only process left. P2 executes for 10 ms from *t* = 20 to *t* = 30, completing its execution. Table 4 illustrates the analysis of the MQAPS method.

**Table 4:** Comparative analysis of MQAPS having dynamic TQ.

Process	Completion time (CT)	Turnaround time (TAT = CT-Arrival Time)	Waiting time (WT = TAT-BT)
P1	15	15 - 0 = 15	15 - 15 = 0
P2	30	30 - 1 = 29	29 - 10 = 19
P2	20	20 - 2 = 18	18 - 5 = 13

Table 5 presents a comparison of the RR and MQAPS methods. The above example is a simple demonstration of how MQAPS works and compares it with traditional RR algorithms.

**Table 5:** Comparison of RR and MQAPS.

Methods	Average turnaround time	Average waiting time
RR	22.33	12.33
MQAPS	20.67	10.67

### Results and discussion

The performance of MQAPS is evaluated by computing the average times *i.e.,* turnaround time and waiting time in milliseconds. The system overhead was also tracked by counting the number of context shifts during process operation. Afterward, the results of MQAPS with several algorithms are compared, such as RPAPS (Iqbal et al., 2023), AN algorithm (Noon, Kalakech & Kadry, 2011), and Multi-Queue Priority Scheduling (Rafi et al., 2018) implemented different scheduling algorithms in different queues, whereas we implemented the same concept in both the queues. MQAPS exhibited superior efficiency, resulting in lower average turnaround and waiting times in a multi-process scenario, as illustrated in the following subsections: Scenario 1, Scenario 2, and Scenario 3. Processes in our study are prioritized on a scale of 1 to 5, structuring each process as a tuple (burst time, priority). All the data required to carry out the experiments to analyze MQAPS under the following three scenarios were generated *via* simulation.

#### Scenario 1

To test how well our algorithm handled smaller processes, we purposely selected processes with relatively short burst periods in the first case. At time *t* = 0, let us consider the arrival of four processes: P1 = [15, 4], P2 = [30, 2], P3 = [45, 3], P4 = [63, 5].

**Figure 2:**
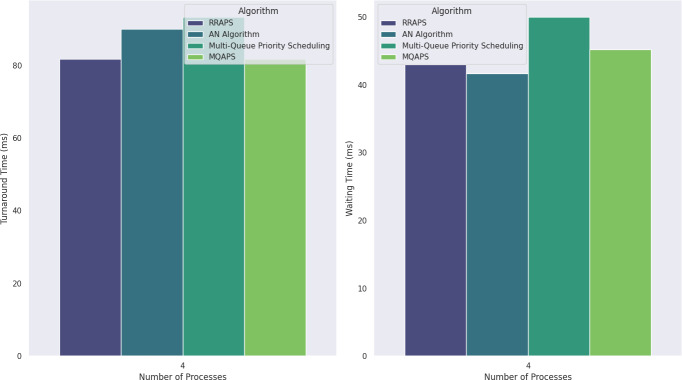
Turnaround and waiting time for four processes are compared.

Table 6 offers a thorough examination of four different scheduling algorithms: MQAPS, AN Algorithm (Noon, Kalakech & Kadry, 2011), RRAPS (Iqbal et al., 2023), and Multi-Queue Priority Scheduling (Rafi et al., 2018). Turnaround time: MQAPS and RRAPS provide the same ideal results at 81.7, beating multi-queue priority scheduling (93.3) and the AN Algorithm (90). This shows that MQAPS controls task completion times while keeping the RRAPS baseline in line. In terms of waiting time, RRAPS is marginally better than MQAPS, scoring 43 instead of 45.2. But both beat multi-queue priority scheduling (50), whereas AN Algorithm shows a better waiting time of 41.7 because it lacks a priority parameter, and that’s why it performs better in terms of waiting time. Figure 2 compares the turnaround and waiting time of these four different methods. Interestingly, MQAPS and RRAPS show the lowest context switching times at 4, indicating how well they switch between tasks. In this case, the performance of RRAPS is marginally better, with less waiting time because of a single queue, compared to MQAPS, having two queues and a threshold check on the SQ.

**Table 6:** Scenario 1: different scheduling algorithms analysis.

Methods	Context switching	Turnaround time (ms)	Waiting time (ms)
RRAPS (Iqbal et al., 2023)	4	81.7	43
AN Algorithm (Omar, Jihad & Hussein, 2021)	5	90	41.7
Multi-Queue Priority Scheduling (Zhao et al., 2019)	5	93.3	50
MQAPS	4	81.7	45.2

 On the other hand, with values of 5, the AN Algorithm and multi-queue priority scheduling necessitate more context switching. The present investigation highlights the competitive performance of MQAPS, demonstrating its ability to manage context switching effectively and yield reduced turnaround and waiting times. This highlights the platform’s potential for process scheduling optimization.

#### Scenario 2

In this case, five processes arrive at different times, *i.e.,* p1 and p2 arrived at time = 0 and time = 3, respectively, where at time 5, p3 arrived, p4 arrived when the time is 6, and p5 time of arrival is 7. Processes BT and priority are given by: P1 = [25, 4], P2 = [38, 1], P3 = [58, 2], P4 = [77, 5], and P5 = [92, 3].

**Figure 3:**
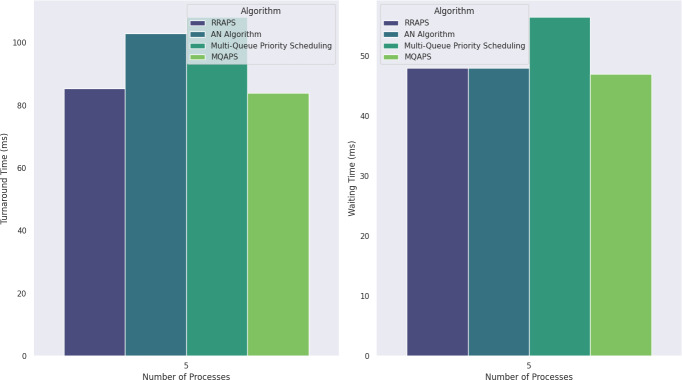
Turnaround and waiting time comparison for five processes.

Table 7 highlights the performance analysis distinctive features of each algorithm. RRAPS displays efficiency with a turnaround and waiting time of 85.4 and 48, respectively. The AN Algorithm is somewhat efficient, with a turnaround time of 103 and a superior waiting time of 44. Multi-queue priority scheduling has much higher processing times (turnaround time: 108.2, waiting time: 56.5). MQAPS stands out with an efficient turnaround time of 84 and a waiting time of 47 as shown in Fig. 3. In terms of context switching, RRAPS has five, AN Algorithm has seven, Multi-Queue Priority Scheduling has eight, and MQAPS has a balanced count of six. MQAPS emerges as a viable method, displaying efficiency in turnaround, waiting time, and context switching, making it a remarkable contender for efficient task execution.

**Table 7:** Scenario 2: results of different algorithmic methods.

Methods	Context switching	Turnaround time (ms)	Waiting time (ms)
RRAPS (Iqbal et al., 2023)	5	85.4	48
AN Algorithm (Omar, Jihad & Hussein, 2021)	7	103	44
Multi-Queue Priority Scheduling (Zhao et al., 2019)	8	108.2	56.5
MQAPS	6	84	47

#### Scenario 3

In Scenario 3, we have 10 processes arrive at different times *i.e.,* P1 and P2 arrived at time = 0, and time = 2 respectively, where at time = 4 P3 arrived, P4 arrived when the time is 5, and P5 time of arrival is 7. P6, P7, and 86 arrive when time is 10, 12, and 15, respectively. P9 arrived at 8, whereas P10 arrived at 13. These 10 processes are P1 = [18, 5], P2 = [39, 2] and P3 = [63, 1], P4 = [77,4], P5 = [99,2]. P6 = [16, 1], P7 = [35, 4] and P8 = [61, 3]. P9 = [9, 4] and P10 = [51, 5]. The evaluation of scenario 3 for each of the algorithms is given in Table 8.

**Table 8:** Scenario 3: evaluation of various algorithmic techniques’ outcomes.

Methods	Context switching	Turnaround time (ms)	Waiting time (ms)
RRAPS (Iqbal et al., 2023)	10	106	81.1
AN Algorithm (Omar, Jihad & Hussein, 2021)	12	120	75
Multi-Queue Priority Scheduling (Zhao et al., 2019)	14	119	79.3
MQAPS	9	98.8	83

**Figure 4:**
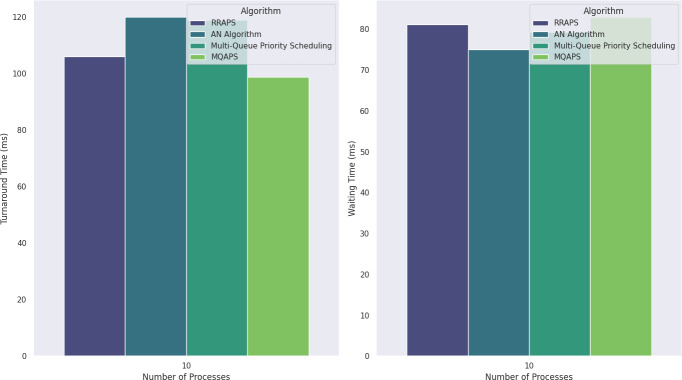
Turnaround and waiting time comparison for 10 processes.

The simulation in Scenario 3 increases processes to ten, each arriving at a distinct time. The analysis in Table 8 identifies MQAPS as the most efficient scheduling algorithm among the choices studied. With a turnaround time of 98.8 as illustrated in Fig. 4, it outperforms RRAPS (106), AN Algorithm (120), and multi-queue priority scheduling (119). MQAPS also outshines the other methods with a competitive waiting time of 83 and minimal context flipping of 9. This demonstrates the efficiency of MQAPS in optimizing process execution, responsiveness, and resource consumption in process scheduling.

**Figure 5:**
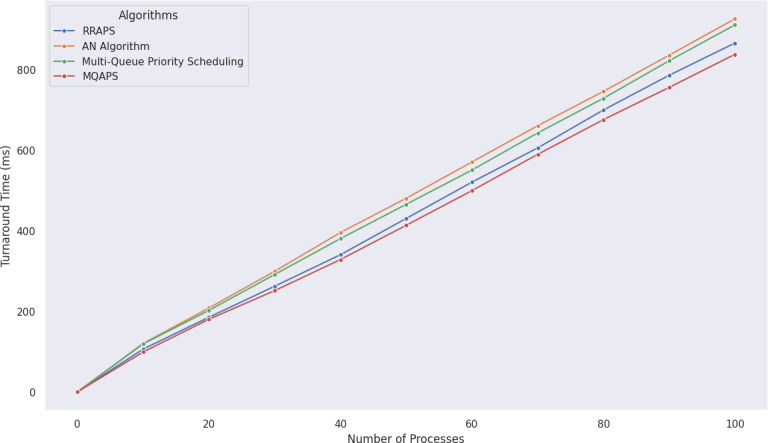
Comparative analysis of turnaround time for 100 processes using various algorithms.

**Figure 6:**
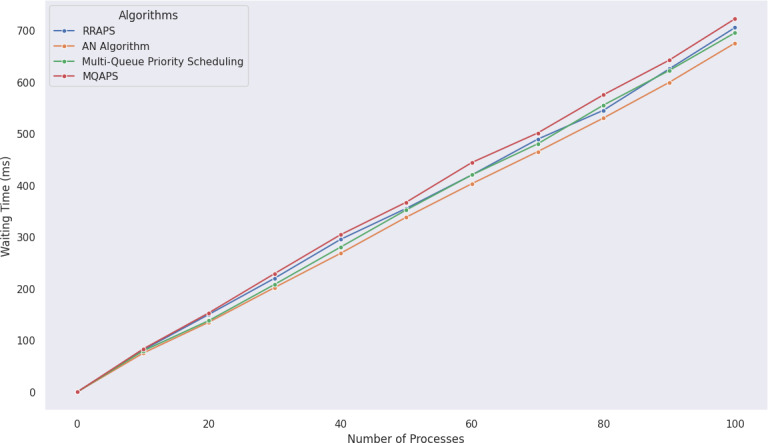
Comparative analysis of waiting time for 100 processes using various algorithms.

MQAPS performance was evaluated using a detailed examination of a dataset containing 100 different processes with varying priority levels and BT. As shown in Figs. 5, 6 and Table 9, the results demonstrated MQAPS as a superior performer, excelling in turnaround time metrics. It is worth noting, however, that the AN Algorithm (Noon, Kalakech & Kadry, 2011) executed admirably in terms of wait time. This distinction results from the AN algorithm’s impartial handling of all processes, which does not discriminate between critical or non-critical things. MQAPS, on the other hand, introduces a secondary queue and priority threshold, resulting in long wait times for low-priority activities while high-priority processes are completed. These findings highlight the advantages and disadvantages of various scheduling strategies. Even though low-priority activities require longer wait times, MQAPS is a valuable method for process scheduling optimization in intricate and dynamic systems. Table 9 presents the statistical analysis demonstrating that MQAPS achieves superior results with large datasets.

### Comparative analysis of resources utilization

**Figure 7:**
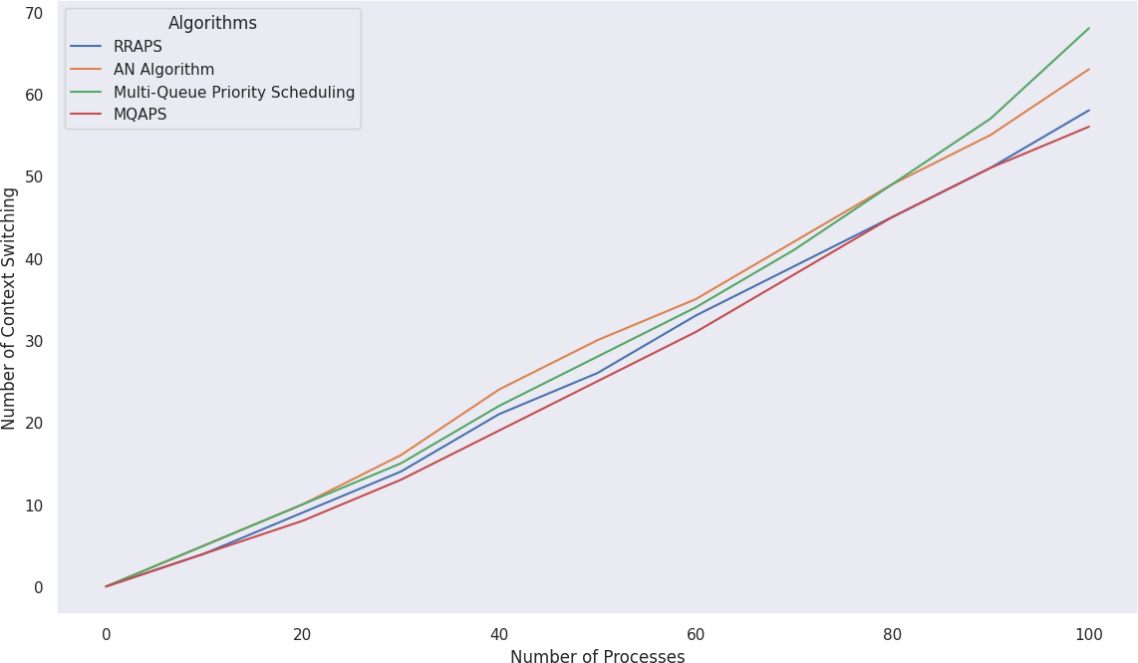
Analyzing context switching among algorithms with 100 processes.

Context switching plays a pivotal role in resource utilization. When a process is switched, its current state is kept and restored to another process state, which adds significant system overhead, as stated in the performance validation section. All this information about the process’s current state and resources used is saved and restored for another process. For MQAPS, 100 processes are simulated, and a full examination of context switching between MQAPS, (Iqbal et al., 2023; Noon, Kalakech & Kadry, 2011), and (Rafi et al., 2018) is shown in Fig. 7 and Table 5. When compared to other scheduling algorithms, MQAPS has less context switching, which implies that resource usage is very effective, there is less system overhead, and the CPU spends more time on execution rather than just switching processes.

**Table 9:** Statistical analysis of 100 processes using different techniques.

Methods	Turnaround time (ms)	Waiting time (ms)	Context switching
RRAPS (Iqbal et al., 2023)	865	705	58
AN Algorithm (Iqbal et al., 2023)	925	675	63
Multi-Queue Priority Scheduling (Zhao et al., 2019)	910	695	68
MQAPS	837	722	56

 In contrast, the MQAPS algorithm, on the other hand, resolves this problem by setting the TQ to the process’s remaining BT. The TQ corresponds to the amount of time required for the execution of a single process while it is in the ready queue, avoiding unnecessary context shifts. By doing this, over-scheduling is avoided, which lowers OS overhead. The method dynamically modifies the time quantum and queues based on process requirements. It minimizes needless context switches and makes it more efficient than round-robin adaptive priority scheduling and conventional round-robin variations.

## Real-World Applications and Integration Potential

### Real-world applications

The MQAPS algorithm is well-suited for real-time systems requiring precise task scheduling and resource management. Potential use cases include:

#### Embedded systems

In IoT and medical devices, MQAPS minimizes latency for high-priority tasks (*e.g.*, heart rate monitoring) while deferring lower-priority operations.

#### Cloud computing

MQAPS optimizes resource allocation by dynamically adjusting time quanta, prioritizing critical workloads and reducing overhead.

#### Autonomous vehicle

MQAPS enhances response times for critical tasks (*e.g.*, collision avoidance) by prioritizing real-time sensor data processing and efficiently managing non-critical tasks.

### Integration potential

The MQAPS algorithm can be seamlessly integrated into widely used operating systems like FreeRTOS and Linux due to its flexible architecture and compatibility with existing scheduling frameworks.

#### FreeRTOS

FreeRTOS currently employs a static priority-based preemptive scheduler. Integrating MQAPS could enhance this by enabling dynamic priority adjustments and adaptive time quanta, improving responsiveness to workload variations. This would optimize task scheduling in embedded and IoT applications where real-time performance is crucial, resulting in lower system overhead and more efficient CPU utilization compared to the existing fixed-priority scheduler.

#### Linux

Linux, especially with the PREEMPT_RT real-time patch, relies on fixed priorities for real-time tasks. Incorporating MQAPS as an extension of the Linux real-time scheduler would provide a more flexible mechanism for managing time-sensitive tasks. Its dual-queue structure and dynamic time quantum adjustments would effectively balance high-priority real-time tasks with lower-priority background processes, enhancing overall system responsiveness and reducing the risk of starvation.

## Limitations of Multi-Queue Adaptive Priority Scheduling

The MQAPS algorithm optimizes task scheduling but faces scalability issues under heavy loads. Increasing task prioritization complexity and dynamic adjustments to time quantum introduces computational overhead, which can degrade efficiency in high-demand real-time systems.

## Conclusions and Future Directions

The dual-tiered design of the MQAPS has better scheduling and effective resource use. By looking at the process’s priority and execution history, the MQAPS specially handles processes. In contrast, a new process is added to the ready queue independent of its execution history or priority. Because of this implementation, CPU time is equitably distributed across all the processes in the ready and secondary queues, preserving process hunger. Efficiency, cost, and system overhead are all balanced by MQAPS. When comparing the results of 100 processes between algorithms, the performance of MQAPS outclasses other methods in terms of turnaround time, context switching, and efficient waiting time. MQAPS is engineered to maintain system equilibrium while expeditiously completing high-priority tasks, augmenting overall throughput. The MQAPS has the potential to address low-priority task starvation, but it may still encounter delays if high-priority tasks continuously arrive and monopolize CPU resources, leading to diminished service for less critical processes.

In the future, we will optimize these limitations and expand the queue to higher levels to incorporate the multi-queue priority scheduling method into the high-level queues. This connection will make MQAPS more flexible and responsive.

## Supplemental Information

10.7717/peerj-cs.2531/supp-1 Supplemental Information 1 Matlab code

10.7717/peerj-cs.2531/supp-2 Supplemental Information 2 Code in text

## Additional Information and Declarations

### Data Availability

The following information was supplied regarding data availability: The code is available in the Supplemental Files.

## References

[ref-1] Abdelhafiz AA (2021). VORR: a new round robin scheduling algorithm. Al-Azhar Bulletin of Science.

[ref-2] Alhaidari F, Balharith TZ (2021). Enhanced round-robin algorithm in the cloud computing environment for optimal task scheduling. Computers.

[ref-3] Ali S, Alshahrani R, Hadadi A, Alghamdi T, Almuhsin F, Sharawy EE (2021). A review on the CPU scheduling algorithms: comparative study. International Journal of Computer Science and Network Security.

[ref-4] Ali KF, Marikal A, Anil Kumar K (2020). A hybrid round robin scheduling mechanism for process management. International Journal of Computer Applications.

[ref-5] Bibu GD, Nwankwo GC (2019). Comparative analysis between first-come-first-serve (FCFS) and shortest-job-first (SJF) scheduling algorithms. International Journal of Computer Science and Mobile Computing.

[ref-6] Chandiramani K, Verma R, Sivagami M (2019). A modified priority preemptive algorithm for CPU scheduling. Procedia Computer Science.

[ref-7] Chen Y, Jia Y (2022). A hierarchical scheduling algorithm for real-time systems using a combination of RR and FCFS scheduling. Journal of Real-Time Systems.

[ref-8] Ghazy N, Abdelkader A, Zaki MS, ElDahshan KA (2022). A new round robin algorithm for task scheduling in real-time system. International Journal of Intelligent Engineering & Systems.

[ref-9] Gupta AK, Mathur P, Travieso-Gonzalez CM, Garg M, Goyal D (2021). ORR: optimized round Robin CPU scheduling algorithm.

[ref-10] Iqbal M, Ullah Z, Ahmad Khan I, Aslam S, Shaheer H, Humayon M, Salahuddin MA, Mehmood A (2023). Optimizing task execution: the impact of dynamic time quantum and priorities on round Robin scheduling. Future Internet.

[ref-11] Kim J, Kim B, Luh H (2019). Analysis of a Markovian feedback queue with multi-class customers and its application to the weighted round-robin queue. Annals of Operations Research.

[ref-12] Lukás˘ K, Mach J (2023). A new FPGA-based task scheduler for real-time systems. Electronics.

[ref-13] Manuel JIC, Baquirin RBM, Guevara KS, Tandingan DR (2019). Fittest job first dynamic round robin (FJFDRR) scheduling algorithm using dual queue and arrival time factor: a comparison. IOP Conference Series: Materials Science and Engineering.

[ref-14] Mostafa SM, Amano H (2020). Dynamic round robin CPU scheduling algorithm based on K-means clustering technique. Applied Sciences.

[ref-15] Niu T, Yang F, Cui M, Huang T (2023). IQQ: infinite Queues in one Queue. ICT Express.

[ref-16] Noon A, Kalakech A, Kadry S (2011). A new round-robin based scheduling algorithm for operating systems: dynamic quantum using the mean average.

[ref-17] Norollah A, Kazemi Z, Sayadi N, Beitollahi H, Fazeli M, Hely D (2021). Efficient scheduling of dependent tasks in many-core real-time system using a hardware scheduler.

[ref-18] Nurmi A, Lindgren P, Szymkowiak T, Hämäläinen TD (2023). AnTiQ: a hardware-accelerated priority queue design with constant time arbitrary element removal.

[ref-19] Omar HK, Jihad KH, Hussein SF (2021). Comparative analysis of the essential CPU scheduling algorithms. Bulletin of Electrical Engineering and Informatics.

[ref-20] Omotehinwa TO (2022). Examining the developments in scheduling algorithms research: a bibliometric approach. Heliyon.

[ref-21] Perez B, Bosque JL (2024). Hardware scheduler for balanced co-execution on integrated GPUs.

[ref-22] Rafi U, Azam Zia M, Razzaq A, Ali S, Saleem MA (2018). Multi-queue priority based algorithm for CPU process scheduling.

[ref-23] Shafi U, Ali Shah M, Wahid A, Abbasi K, Javaid Q, Asghar MN, Haider M (2020). A novel amended dynamic round robin scheduling algorithm for timeshared systems. The International Arab Journal of Information Technology.

[ref-24] Sharma C, Sharma S, Kautish S, Alsallami SAM, Khalil EM, Mohamed AW (2022). A new median-average round Robin scheduling algorithm: an optimal approach for reducing turnaround and waiting time. Alexandria Engineering Journal.

[ref-25] Vecliuc D-D, Leon F, Logofătu D (2022). A comparison between task distribution strategies for load balancing using a multi-agent system. Computation.

[ref-26] Xu J, Shi H, Chen Y (2023). Efficient tasks scheduling in multicore systems integrated with hardware accelerators. The Journal of Supercomputing.

[ref-27] Zhang Z, Wang Z, Li S, Wang F (2023). A hierarchical scheduling algorithm for real-time systems. Journal of Computer Science and Technology.

[ref-28] Zhao Y, Suo K, Wu X, Rao J, Wu S, Jin H (2019). Preemptive multi-queue fair queuing.

